# Special Issue “Neurobiology of Protein Synuclein”

**DOI:** 10.3390/ijms25063223

**Published:** 2024-03-12

**Authors:** Mattia Toni

**Affiliations:** Department of Biology and Biotechnologies “Charles Darwin”, Sapienza University, 00161 Rome, Italy; mattia.toni@uniroma1.it

## 1. Introduction

Synucleins are a family of proteins consisting of α, β, and γ synuclein (syn). The first syn was discovered in the fish *Torpedo californica* [1], and subsequently syn isoforms were detected in all classes of vertebrates, although there may be variations in the number and type of synucleins expressed in the different species [2].

Among the three proteins, α-syn appears to be particularly studied for its involvement in the onset of serious neurodegenerative pathologies such as Parkinson’s disease (PD), dementia with Lewy bodies, PD dementia, multiple system atrophy, and other less well-characterised neuroaxonal dystrophies [3]. These pathologies are collectively called synucleinopathies since they are characterised by amyloidogenic aggregates of hyperphosphorylated α-syn rich in β-sheets that can occur in neurons and glia cells of the central and peripheral nervous system [4,5,6,7]. The direct involvement of α-syn in neurodegenerative diseases is also demonstrated by the discovery of mutations in the α-syn gene SNCA (A30P, E46K, H50Q, G51D, and A53T) resulting in autosomal-dominant PD [8,9,10,11,12,13].

The α-syn is a fascinating protein, characterised by prion-like mechanisms as it can exist either in a soluble natively unfolded form or undergo conformational conversions in a β-sheet-rich protein prone to aggregate, forming amyloidogenic α-syn. Moreover, the presence of amyloidogenic α-syn can function as a template to guide the conversion of the physiologically unfolded α-syn to the β sheet-rich protein [14]. The progression and spread of the synucleinopathies are believed to result from the ability of pathogenic α-syn to transmit from cell to cell allowing the propagation of prion-like proteins along neuroanatomical traits. It has been proposed that PD pathology can spread with a prion-like mechanism through the olfactory and vagal systems to the *substantia nigra* [15].

Given the peculiarity of synucleins, a large amount of information is still needed to fully understand their physiological functions and the mechanisms underlying the onset and propagation of synucleinopathies. Further studies on syn function in different neuronal populations and cellular districts are needed. Moreover, since the function of these proteins depends on the interaction with other molecules, it is necessary to acquire complete knowledge of syn-interacting proteins and the effect of these interactions. Furthermore, it is important to investigate the role and involvement of homeostasis mechanisms, including autophagy, since synucleinopathies are associated with the accumulation of α-syn aggregates that the cell cannot remove. Given the wide complexity of the nervous system and the different roles of specific neuronal populations, the effect of the formation of α-syn aggregates must be studied in different neuronal populations to understand the causes of the symptoms of patients suffering from synucleinopathies. On the other hand, the close association between neurodegenerative diseases and neuroinflammation requires investigating the involvement of synucleins in these processes. The neurodegenerative effects of synucleinopathies arise from the ability of the disease to spread and progress. The comprehension of the mechanisms that underlie the cell-to-cell transmission of misfolded synucleins and the formulation of novel strategies to prevent the onset of synucleinopathies, including the creation of targeted vaccines, are of paramount importance. Interesting studies that address these topics are presented in this Special Issue.

## 2. An Overview of the Published Articles

The work of Bonaccorsi et al. [16] analyses synuclein expression in *Xenopus laevis*, a model organism in various scientific research fields including neuroscience [17,18,19,20,21], using Western blot, real-time PCR, and immunohistochemistry. The expression of α, β, and γ syn in the nervous system makes *X. laevis* a suitable candidate as a study model for synucleinopathies. Moreover, its α-syn possesses a threonine instead of alanine in position 53 of the human protein, and this substitution may be biologically relevant since the A53T mutation in humans is linked to PD. Circular dichroism was used to analyse the effect of this substitution on the secondary structure of *Xenopus* α-syn.

The term “synuclein” was coined because early observations localised the protein at both presynaptic and nuclear levels [1]. Most studies have focused mainly on α-syn localised in the cytoplasm, where amyloid inclusions can form, and at presynaptic terminals where the protein has been involved in the regulation of neurotransmitter release through the interaction with synaptic vesicles [22]. The α-syn nuclear localisation has been confirmed in several study models such as cultured cells [23,24,25,26], α-syn transgenic *Drosophila* [27], and mice [28,29,30]. Furthermore, intranuclear α-syn inclusions have been observed in neurons in multiple system atrophy [31] and PD patients [28]. Studies show that nuclear α-syn interacts with DNA [32,33] and histones [34], modulating their acetylation levels [29,34,35] and promoting neurotoxicity [29]. Nuclear α-syn influences DNA repair [33] and the regulation of gene transcription [36,37]. The formation of intranuclear inclusions can be facilitated by α-syn mutations (e.g., A30P, A53T, or G51D) or increased expression of wildtype α-syn [28,29,38,39,40]. Specific mutations can increase the binding of the protein to DNA compared to endogenous α-syn [41]. Furthermore, α-syn nuclear localisation affects the cognitive and motor behaviour of mice by inducing DNA damage and the abnormal cell cycle of hippocampal neurons [42]. Interestingly, studies have underscored a correlation between oxidative stress and nuclear α-syn, as oxidative stress can induce the nuclear translocation of α-syn, and this increases a cell’s vulnerability to oxidative stress [43,44]. The impact of α-syn nuclear localisation has been further explored in mouse embryonic fibroblasts by Ho and colleagues [45], as detailed in this Special Issue. Their findings underscore the role of nuclear α-syn in inducing oxidative stress-related cytotoxicity in mouse embryonic fibroblasts. Nuclear α-syn significantly impacts cell fate, resulting in alterations in ribosomal biogenesis, nucleolar segmentation, and apoptosis.

Protein functions strictly depend on their interactions with other molecules. The identification of α-syn-interacting proteins is important for understanding their functions and physiopathological roles. Studies aimed at analysing the interactome of α-syn oligomers have identified important proteins capable of interacting with α-syn including VAMP, NKA, CaMKII-G, TUBB3, 14-3-3 protein, BASP1, MAP-1B, VDAC, Hsp60, V-ATPase, and CaMKII-G [46]. The work of Mativ and collaborators published in this Special Issue further investigates this topic by highlighting that NOS1AP interacts with α-syn and aggregates in yeast and mammalian cells [47]. The NOS1AP (CAPON) gene encodes a cytosolic protein that binds neuronal nitric oxide synthase and regulates its activity [48]. The results support the ability of NOS1AP to interact and co-aggregate with α-syn suggesting a molecular mechanism underlying the coexistence of schizophrenia and PD.

Autophagy is an essential process for maintaining cellular and neuronal homeostasis [49] because it allows the isolation and degradation of dysfunctional organelles and misfolded proteins, including α-syn [50]. This Special Issue includes research by Dhanushkodi and colleagues, which explores the impact of disrupting autophagy by silencing the ATP13A2 (PARK9) gene, an autophagy-related protein, in *Drosophila* expressing wildtype or mutated α-syn. Their results suggest that the reduction in ATP13A2 contributes to increase the aggregation of mutant A53T-α-syn with a concomitant increase in p62 levels, causing enhanced dopaminergic neuron loss and exacerbating the non-motor phenotype.

Among the synucleinopathies, PD is one of the most widespread neurodegenerative pathologies that is characterised by the aggregation and accumulation of α-syn and the progressive loss of dopaminergic neurons in the *substantia nigra*. This alters the correct functioning of the *substantia nigra pars compacta*–striatum system causing cardinal motor symptoms including resting tremor, rigidity, bradykinesia, and postural instability [51]. The work of Cai and collaborators [52] published in this Special Issue explores the causal role of α-syn in the development of forelimb and cranial fine movement deficits within the *substantia nigra*–dorsolateral striatum dopaminergic pathway. The study uses transgenic mice expressing the A53T-α-syn in the dopaminergic neurons of the *substantia nigra* and involves the injection of α-syn fibrils into the dorsal striatum or *substantia nigra*.

Neuroinflammatory processes participate in most neurodegenerative diseases, including synucleinopathies. Glia cells play a key role in neuroinflammatory processes, being able to assume both pro-inflammatory and neuroprotective phenotypes. Although the main purpose of neuroinflammation is to protect the nervous system by maintaining its homeostasis through promoting tissue repair and removing cellular debris, as well as inhibiting or removing various pathogens, it can be detrimental when it occurs for prolonged periods and can lead to neurodegeneration [53]. Evidence shows a correlation between α-syn, microglia, and neuroinflammation. This Special Issue contains two reviews conducted by Lyra and collaborators [54] and Forloni [55], which focus on the involvement of α-syn in neurodegeneration and inflammation.

The identification of the molecules that favour or counteract the transmission of pathogenic α-syn is fundamental to understanding the mechanisms underlying the onset and spread of synucleinopathies. This Special Issue contains three different studies that obtain important results on this topic. The work of De La Cruz and collaborators shows that the loss of MhcII leads to dysfunctional responses in CNS-resident microglia and astrocytes to α-syn prion seeds [56]. Specifically, in MhcII^−/−^ mice injected with pathogenic α-syn preformed fibrils, the absence of MhcII reduced the lifespan, accelerated the seeding of α-syn pathology, and reduced the p62 accumulation [56]. The study of Kang and collaborators shows that neuronal ApoE deficiency attenuates both α-syn uptake and release and enhance chaperone-mediated autophagy [57]. Moreover, the study also shows that α-syn propagation is attenuated in ApoE-knockout mice injected with pre-formed mouse α-syn fibrils.

The production of monoclonal antibodies specific to α-syn through vaccine could be effective in counteracting the onset and progression of synucleinopathies by reducing or inhibiting the accumulation of aggregated α-syn in the brain. Evidence has shown that antibodies generated after active and passive vaccinations can inhibit the spread of pathological α-syn in the extracellular space and prevent or inhibit diseases in relevant animal models. The brief report by Zagorski and collaborators [58] published in this Special Issue developed a recombinant protein-based MultiTEP vaccine and tested its immunogenicity in young and aged D-line mice.

The Special Issue concludes with an interesting work by Halbgebauer and collaborators that assesses brain-specific β-syn as a novel blood biomarker of synaptic damage. The results show that plasma β-synuclein are significantly increased after polytrauma compared to controls. The work demonstrates that plasma concentrations of β-syn, together with NfL and GFAP, have a good predictive performance for fatal outcome in polytrauma.

## 3. Conclusions

This Special Issue encompasses studies that explore fundamental aspects of synucleins, areas in which research should concentrate to acquire the essential information needed to combat synucleinopathies.
